# Clinical factors associated with peri-implantitis and the culture-based microbiological profile of peri-implantitis cases: a retrospective matched case-control study

**DOI:** 10.3389/fcimb.2026.1848675

**Published:** 2026-09-24

**Authors:** Yan-Jie Chai, Jia-Qing Sun

**Affiliations:** Department of Stomatology, Tongxiang First People’s Hospital, Tongxiang, Zhejiang, China

**Keywords:** antimicrobial susceptibility, case-only culture profile, clinical indicators, dental implants, matched case-control study, peri-implantitis

## Abstract

**Background:**

Peri-implantitis is a biofilm-associated inflammatory disease that can compromise peri-implant tissue stability and implant survival. This study separately examined clinical indicators in a matched case-control analysis and described culture findings among peri-implantitis cases; the microbiological component was not designed to compare disease with health.

**Methods:**

This retrospective matched case-control study included consecutive adults receiving dental implant treatment between June 2021 and June 2025. Cases met the 2017 World Workshop case definition for peri-implantitis (n = 128), whereas controls without peri-implantitis were selected at a 1:2 ratio (n = 256) by matching on index year and observation time. Microbiological sampling was performed only in cases at disease presentation. All 128 cases were retained in the clinical analyses regardless of culture result; organism-distribution and susceptibility summaries were limited to culture-positive episodes. Clinical associations were evaluated using a *post hoc* exploratory multivariable logistic regression model.

**Results:**

Among the 128 peri-implantitis cases, bacterial growth was recovered from 112 episodes (87.5%), whereas 16 episodes (12.5%) were culture-negative. No single organism predominated; the four most frequently recovered taxa each occurred in 19.6%-28.6% of culture-positive episodes. These frequencies are descriptive of cases only. In the matched clinical comparison, history of periodontitis was associated with peri-implantitis (adjusted OR 1.93, 95% CI 1.25-2.99). The larger estimate for immune-related disorders was imprecise (adjusted OR 5.84, 95% CI 1.67-20.47). Anodized implant surface, longer surgery duration, pre-index occlusal overload, higher pre-index plaque index, and fewer maintenance visits were also retained in the exploratory model.

**Conclusions:**

Culture results describe viable organisms recovered from peri-implantitis cases under the study conditions and do not permit comparison with healthy implants. History of periodontitis was associated with peri-implantitis (adjusted OR 1.93, 95% CI 1.25-2.99); other model estimates remain exploratory because the covariates were selected *post hoc*, some exposures were sparse, and the analysis was not validated.

## 1 Introduction

Dental implant therapy is widely adopted for the rehabilitation of partial and complete edentulism; however, infectious complications remain a clinically relevant cause of morbidity and may compromise osseointegration, peri-implant tissue stability, and long-term implant survival (Galarraga-Vinueza et al., 2025). Contemporary epidemiologic syntheses indicate that peri-implant diseases affect a substantial proportion of implant-treated patients, underscoring the need for effective prevention, early detection, and risk-oriented follow-up pathways (Heitz-Mayfield, 2024). Clinical practice guidelines and consensus frameworks emphasize that peri-implant conditions are biofilm-driven and that patient- and site-level risk indicators should inform individualized supportive care and treatment decisions (West et al., 2024; Fiorellini et al., 2025).

Peri-implantitis develops within a complex ecological niche in which the biofilm is shaped by local tissue architecture, implant-abutment design and surface features, and host immune responses. Microbiome investigations and systematic reviews indicate compositional differences across peri-implant health, mucositis, and peri-implantitis, including enrichment of anaerobe-associated taxa and periodontitis-linked organisms in disease states (Chun Giok and Menon, 2023; Espinoza-Arrue et al., 2025). Implant surface characteristics may also influence microbial adhesion and disease-associated community structure (Sinjab et al., 2024; Zhou et al., 2026). In routine care, microbiological testing is generally performed only when disease is clinically suspected; consequently, culture-based series commonly describe diseased sites without a healthy microbiological comparator. Retrospectively recorded procedural and maintenance variables are not uniformly modifiable, and their temporal relationship to disease must be established before they can be interpreted as candidate clinical indicators. Translation of antimicrobial implant surfaces into routine care also remains constrained by evidentiary, regulatory, manufacturing, and implementation barriers (Borges et al., 2024).

Few routine-care studies have jointly reported a case-only culture profile of clinically diagnosed peri-implantitis and an index-aligned matched clinical comparison while maintaining clear analytic separation between these components. This study therefore had two distinct aims: (1) to describe culture yield, recovered organisms, and antimicrobial susceptibility patterns among peri-implantitis cases only; and (2) to examine exploratory associations between peri-implantitis and patient-, implant-, procedural-, occlusal-, and maintenance-related indicators. Because controls were not microbiologically sampled, no organism-specific association with disease status was estimated.

## 2 Methods

### 2.1 Study design

This retrospective matched case-control study enrolled consecutive patients who underwent dental implant placement and/or implant restoration at our institution between June 2021 and June 2025. The report was prepared in accordance with the STROBE recommendations for observational studies (von Elm et al., 2007). Patients with peri-implantitis were assigned to the case group (n = 128), and controls without peri-implantitis were selected at a 1:2 ratio (n = 256). Eligibility criteria and exclusions were applied before outcome-based sampling. Only the first eligible implant episode per patient was retained. The study protocol was reviewed and approved by the Institutional Ethics Committee of Tongxiang First People’s Hospital. All records were de-identified before analysis, and the study was conducted in accordance with the Declaration of Helsinki.

### 2.2 Study population and grouping

Cases were restricted to peri-implantitis; patients with peri-implant mucositis without progressive bone loss were not included. Case ascertainment followed the 2017 World Workshop consensus and diagnostic case definitions (Berglundh et al., 2018). A case required clinical peri-implant inflammation (bleeding and/or suppuration on probing), radiographic bone loss beyond initial post-loading remodeling, and increased probing depth relative to prior post-restoration measurements. When previous radiographs or probing measurements were unavailable, the consensus surrogate threshold (radiographic bone level ≥3 mm together with bleeding on probing and probing depth ≥6 mm) was applied (Renvert et al., 2018). The index date was the first encounter at which peri-implantitis was established and recorded.

Controls were eligible implant-treated patients who remained free of peri-implantitis through the corresponding reference date. Of 936 eligible controls before matching, 256 were selected at a 1:2 ratio; 680 remained unmatched and no case was discarded for lack of a suitable control. Matching was used only to align treatment calendar time and the opportunity for disease ascertainment (observation time). The score model therefore included index year and observation time, followed by nearest-neighbor matching without replacement using a caliper of 0.2 standard deviations of the logit. Clinical variables were intentionally not balanced by design because they were exposures of interest; they were evaluated in the subsequent regression model. Accordingly, this procedure should not be interpreted as eliminating clinical confounding, and residual confounding remains possible.

### 2.3 Case-only microbiological sampling, culture, and descriptive profiling

Microbiological sampling was performed only for peri-implantitis cases at the index presentation. The sampling field was isolated with cotton rolls. Supragingival plaque was gently removed, and the site was air-dried without applying an antiseptic to the sampled pocket. Sterile paper points were inserted to the base of the deepest peri-implant pocket for 30 seconds. Visible exudate or purulent material was collected with a separate sterile swab when present. Specimens were placed immediately in pre-reduced anaerobic transport medium, maintained at controlled room temperature, delivered to the clinical microbiology laboratory within 2 hours, and not frozen. Samples exceeding the laboratory transport-time acceptance window were rejected. Aerobic and facultative organisms were cultured on 5% sheep-blood agar and chocolate agar at 35–37 °C in 5% CO2 for 24–48 hours; MacConkey agar was used when Gram-negative bacilli were suspected. Obligate anaerobes were inoculated onto Brucella blood agar supplemented with hemin and vitamin K1 and incubated at 35–37 °C under anaerobic conditions for 5–7 days. Following incubation, all morphologically distinct colony types recovered from the primary plates were evaluated: one representative colony per morphotype and specimen was subcultured to purity, rather than every colony within confluent growth being tested individually. Initial characterization used colony morphology, Gram staining, and organism-appropriate phenotypic and biochemical tests, including catalase, oxidase, coagulase, and commercial biochemical panels when required. All pure isolates were subsequently analyzed by MALDI-TOF MS using a Microflex LT instrument with MALDI Biotyper software version 4.1 (Bruker Daltonics, Bremen, Germany). A Biotyper score ≥2.00 was accepted for species-level identification, a score of 1.70–1.99 supported genus-level identification, and a score <1.70 was considered unreliable. Isolates below the species-level threshold were re-extracted and retested; persistent genus-only results were reported at the highest taxonomic level supported by concordant phenotypic and biochemical findings. A written workflow for sampling, transport, culture, identification, acceptance criteria, and daily quality control was applied throughout the study period; changes in platform or breakpoint edition were documented in the laboratory information system (Miller et al., 2024). Accordingly, the microbiological profile represents only viable, cultivable organisms recovered under the specified sampling and incubation conditions and does not represent the complete peri-implant microbiota.

Antimicrobial susceptibility testing was performed on clinically relevant isolates using disk diffusion or broth microdilution, as appropriate for the organism, with a 0.5 McFarland inoculum and organism-specific media and atmospheric conditions. Zone diameters and minimum inhibitory concentrations were interpreted using the CLSI M100 edition in force at the time of testing (Clinical and Laboratory Standards Institute, 2025). Cefoxitin screening and/or oxacillin minimum inhibitory concentration was used to classify methicillin-resistant *Staphylococcus aureus*. Quality-control strains were included according to CLSI requirements. Mixed infection was defined *a priori* as recovery of at least two distinct bacterial species from the specimen set collected during one index episode. Multidrug resistance was defined as acquired non-susceptibility to at least one agent in three or more antimicrobial categories (Magiorakos et al., 2012).

### 2.4 Data collection and quality control

Data were retrospectively extracted from electronic medical records, implant treatment records, radiographic archives, maintenance records, and the laboratory information system. Demographic characteristics, systemic conditions, medication exposure, and other clinical variables were collected from information documented before the index date for cases or the corresponding reference date for controls. Fixed baseline characteristics and comorbidities were defined according to records available at or before implant placement. Immune-related disorders were defined as documented autoimmune or inflammatory disorders or primary immune disorders present before implantation, whereas exposure to immunosuppressive medications was recorded separately. Because immune-related disorders were uncommon and clinically heterogeneous, this variable was treated as exploratory. Systemic antibiotic exposure was classified only when it occurred before the index/reference date; antibiotic courses initiated at the diagnostic encounter or thereafter were excluded from etiologic analyses to reduce potential reverse-causation bias.

Implant-related and operative variables were retrospectively abstracted from the original implant procedure records and therefore preceded the occurrence of peri-implantitis. Postoperative variables were temporally aligned with the index/reference date. Maintenance frequency was calculated as the annualized number of completed supportive peri-implant care visits before that date, and plaque index was defined as the most recent documented value obtained before that date. Measurements recorded at or after the diagnosis of peri-implantitis were excluded. Occlusal overload was considered present only when it had been documented before the index/reference date on the basis of excessive or premature contacts detected using articulating paper together with at least one supporting clinical finding, such as repeated screw loosening, prosthetic wear or fracture, or clinician-documented heavy occlusal contact. Occlusal adjustment performed in response to the index presentation was not classified as a pre-disease exposure. The same temporal definitions were applied to cases and controls.

Data were independently abstracted by two investigators using prespecified variable definitions and a standardized data-collection form. Extracted data were cross-checked, and disagreements were resolved through review of the original records and consensus. Missingness was assessed for selected candidate and modeled variables, quantified overall, and compared between cases and controls. Multiple imputation by chained equations was performed to generate 20 imputed datasets. Predictive mean matching was used for continuous variables, whereas binary or multinomial logistic regression models were used for categorical variables, as appropriate. The imputation model included outcome status, matching variables, and all candidate covariates, and regression estimates were combined using Rubin’s rules. Descriptive summaries for variables with missing observations were averaged across the imputed datasets; displayed categorical counts represent the corresponding rounded values. Complete-case and missing-indicator analyses were conducted as sensitivity analyses.

### 2.5 Statistical analysis

All analyses were performed using IBM SPSS Statistics version 28.0 and R version 4.3.2. Continuous variables were summarized as mean ± standard deviation or median [interquartile range], according to their distributions, and categorical variables were summarized as n (%). Between-group comparisons used independent-samples t tests, Mann-Whitney U tests, Pearson chi-square tests, or Fisher exact tests, as appropriate. Matching aligned index year and observation time but was not assumed to control all clinical confounding; standardized mean differences were reported for the two matching variables. Microbiological outcomes were descriptive and limited to cases. All 128 cases remained in the matched clinical analyses, including the 16 culture-negative episodes. Culture yield was defined as the proportion of the 128 index case specimens with any recoverable bacterial growth. Organism-distribution and susceptibility summaries used only culture-positive episodes because no organisms were recovered from culture-negative specimens. No inferential comparison was performed for the microbiological profile, and no organism-disease association was estimated because controls were not microbiologically sampled. A *post hoc* exploratory unconditional multivariable logistic regression model estimated associations with peri-implantitis as adjusted odds ratios (ORs) with 95% confidence intervals (CIs). Only measurements preceding the index/reference date were eligible for etiologic interpretation. For this *post hoc* model, implant surface was coded as anodized versus non-anodized, with SLA and machined/other surfaces combined in the reference category. The retained model included seven temporally eligible indicators and 128 case events, corresponding to 18.3 events per parameter. The covariate set was not prespecified; therefore, the estimates and confidence intervals do not incorporate variable-selection uncertainty and should not be interpreted as confirmatory or causal. Antibiotic use or occlusal adjustment initiated in response to disease was excluded from etiologic interpretation. No *a priori* sample-size calculation was performed because the study used all eligible cases in a fixed historical cohort. Multicollinearity was assessed using the variance inflation factor. The regression analysis did not explicitly model dependence within matched sets. No internal or external validation was performed, and the model was not presented as a clinical prediction tool. All tests were two-sided, with P < 0.05 indicating statistical significance.

## 3 Results

### 3.1 Study population and selection

During the study period, 1,120 consecutive adults receiving implant placement and/or restoration were screened. Fifty-six were excluded: non-institutional treatment or nonstandard records (n = 14), incomplete key data (n = 16), inadequate follow-up (n = 12), non-bacterial/non-inflammatory peri-implant conditions (n = 6), major immunosuppression or prior head-and-neck radiotherapy (n = 3), acute oral infection at implantation (n = 2), and duplicate episodes (n = 3). Among 1,064 eligible patients, 128 met the peri-implantitis definition and 936 were eligible controls. Matching retained all 128 cases and selected 256 controls; 680 eligible controls were not selected. The final analytic cohort therefore comprised 384 patients (**Figure 1**).

**Figure 1:**
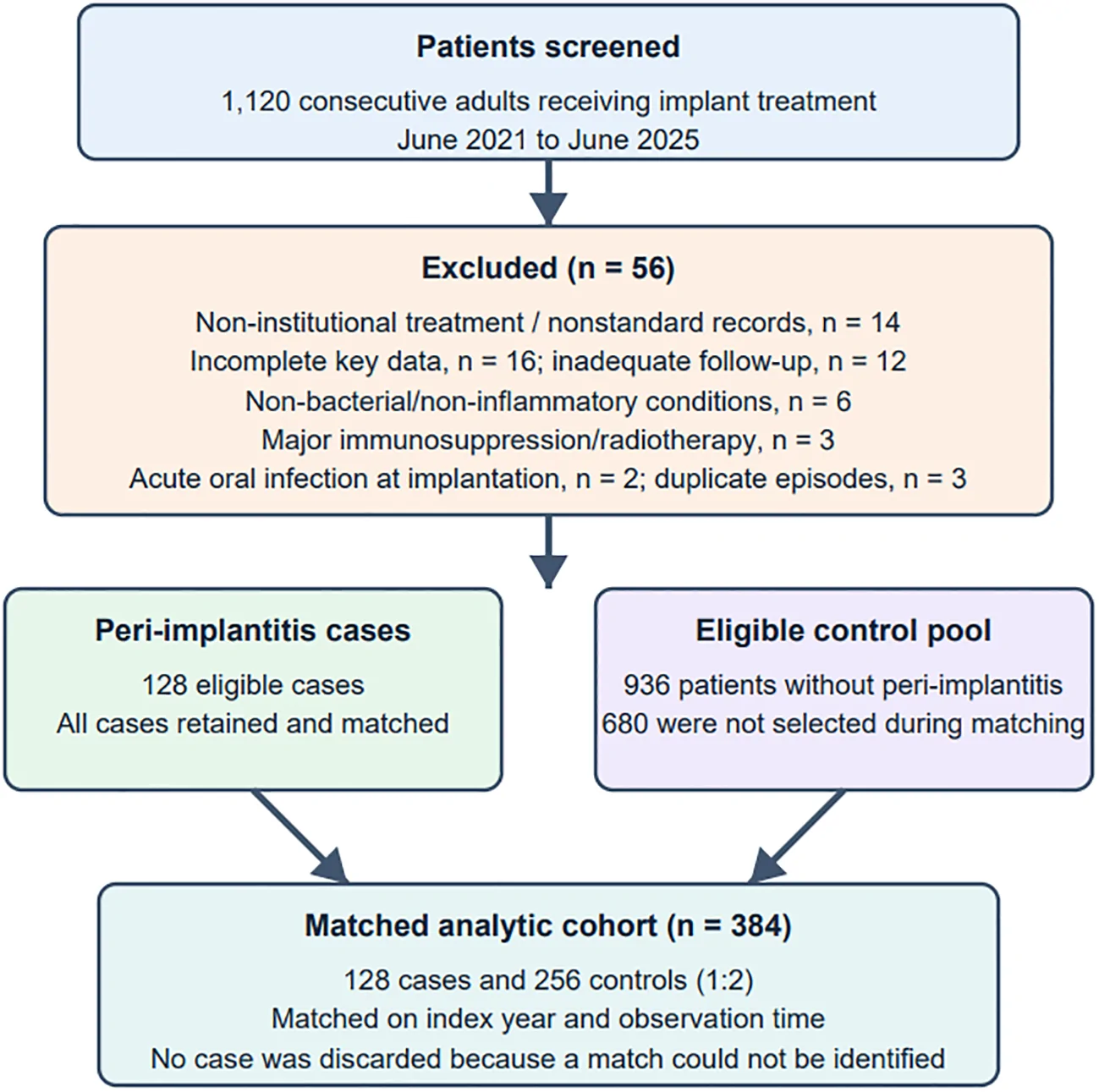
STROBE-style participant selection and matching flow. Of 1,120 screened patients, 56 were excluded; 128 peri-implantitis cases and 936 eligible controls remained. All cases were matched to 256 controls, while 680 eligible controls were not selected.

### 3.2 Matching performance and balance diagnostics

Before matching, observation time was longer in the eligible control pool than in cases (19.0 ± 8.5 vs 14.2 ± 6.1 months; P < 0.001; SMD = 0.649). After matching, index-year distributions were identical and observation time was aligned (14.3 ± 6.0 vs 14.2 ± 6.1 months; P = 0.879; SMD = 0.017). These diagnostics confirm balance only for the two prespecified matching variables; clinical covariates were not expected to be balanced by this design step (**Supplementary Table 1**).

### 3.3 Baseline characteristics

Mean age was similar (51.64 ± 9.57 vs 52.21 ± 9.41 years; t = -0.56, P = 0.577), as were the proportions of male patients (53.9% vs 52.7%; chi-square = 0.05, P = 0.828) and BMI (24.51 ± 3.27 vs 24.29 ± 3.05 kg/m²; t = 0.63, P = 0.532). Smoking status showed a borderline between-group difference (chi-square = 5.36, P = 0.069), with a higher proportion of current smokers among cases. Current alcohol use was also more frequent among cases, although the difference did not reach statistical significance (21.9% vs 14.5%; chi-square = 3.34, P = 0.068). Diabetes mellitus (26.6% vs 11.7%; chi-square = 13.54, P < 0.001), history of periodontitis (44.5% vs 27.3%; chi-square = 11.39, P < 0.001), and immune-related disorders (7.8% vs 1.2%; Fisher exact P = 0.001) were more frequent among cases, whereas hypertension was similar between groups (P = 0.466). Pre-index systemic antibiotic exposure was higher among cases (39.8% vs 23.4%; chi-square = 11.18, P < 0.001), whereas corticosteroid and immunosuppressant use did not differ significantly (**Table 1**).

**Table 1:** Demographic, behavioral, systemic, and pre-index medication variables.

Variable	Cases (n=128)	Controls (n=256)	Test statistic	P value
Age, years	51.64 ± 9.57	52.21 ± 9.41	t=-0.56	0.577
Male sex, n (%)	69 (53.9)	135 (52.7)	χ²=0.05	0.828
BMI, kg/m²	24.51 ± 3.27	24.29 ± 3.05	t=0.63	0.532
Smoking status, n (%)	Never 67 (52.3); Former 28 (21.9); Current 33 (25.8)	Never 156 (60.9); Former 59 (23.0); Current 41 (16.0)	χ²=5.36	0.069
Current alcohol use, n (%)	28 (21.9)	37 (14.5)	χ²=3.34	0.068
Diabetes mellitus, n (%)	34 (26.6)	30 (11.7)	χ²=13.54	<0.001
History of periodontitis, n (%)	57 (44.5)	70 (27.3)	χ²=11.39	<0.001
Immune-related disorders, n (%)	10 (7.8)	3 (1.2)	Fisher exact	0.001
Hypertension, n (%)	38 (29.7)	67 (26.2)	χ²=0.53	0.466
Pre-index systemic antibiotic exposure, n (%)	51 (39.8)	60 (23.4)	χ²=11.18	<0.001
Systemic corticosteroids, n (%)	11 (8.6)	12 (4.7)	Fisher exact	0.170
Immunosuppressive agents, n (%)	6 (4.7)	6 (2.3)	Fisher exact	0.225

BMI, body mass index. Immune-related disorders were heterogeneous and uncommon (10 cases and 3 controls); the corresponding estimate should be interpreted cautiously. Antibiotic exposure denotes pre-index use only and excludes treatment initiated at disease presentation. For variables with missing observations, descriptive summaries were averaged across the 20 imputed datasets; displayed categorical counts are rounded to the nearest whole number.

### 3.4 Oral, implant, and maintenance profiles

Several oral and implant-related variables differed between groups after matching. Implant jaw distribution was similar (maxilla: 57.0% vs 48.8%; χ² = 2.30, P = 0.129), whereas posterior-region implants were more common in cases (72.7% vs 62.5%; χ² = 3.92, P = 0.048). Implant diameter was slightly larger (4.20 ± 0.38 vs 4.11 ± 0.36 mm; t = 2.15, P = 0.033) and implant length slightly shorter in cases (10.81 ± 1.19 vs 11.13 ± 1.19 mm; t = −2.51, P = 0.013). Surface-category distributions differed (χ² = 13.29, P = 0.001). Immediate placement was less frequent among cases (20.3% vs 30.5%; χ² = 4.46, P = 0.035), and surgery duration was longer (66.99 ± 20.45 vs 59.28 ± 16.93 min; t = 3.68, P < 0.001). Prosthesis type also differed (χ² = 13.21, P = 0.001). Occlusal overload was more frequent in cases (18.8% vs 7.0%; χ² = 12.03, P < 0.001), and occlusal adjustment was higher (P = 0.041). Cases had fewer maintenance visits (Z = −3.83, P < 0.001) and higher plaque index values (t = 7.46, P < 0.001) (**Table 2**).

**Table 2:** Oral, implant, surgical, prosthetic, and temporally aligned maintenance variables.

Variable	Cases (n=128)	Controls (n=256)	Test statistic	P value
Implant location: maxilla, n (%)	73 (57.0)	125 (48.8)	χ²=2.30	0.129
Implant region: posterior, n (%)	93 (72.7)	160 (62.5)	χ²=3.92	0.048
Implant diameter, mm	4.20 ± 0.38	4.11 ± 0.36	t=2.15	0.033
Implant length, mm	10.81 ± 1.19	11.13 ± 1.19	t=-2.51	0.013
Implant surface, n (%)	SLA 80 (62.5); Anodized 43 (33.6); Machined/other 5 (3.9)	SLA 181 (70.7); Anodized 48 (18.8); Machined/other 27 (10.5)	χ²=13.29	0.001
Immediate implant placement, n (%)	26 (20.3)	78 (30.5)	χ²=4.46	0.035
One-stage surgery, n (%)	86 (67.2)	166 (64.8)	χ²=0.21	0.649
Bone augmentation, n (%)	40 (31.2)	63 (24.6)	χ²=1.92	0.166
Sinus floor elevation, n (%)	18 (14.1)	26 (10.2)	χ²=1.28	0.257
Surgery duration, min	66.99 ± 20.45	59.28 ± 16.93	t=3.68	<0.001
Loading protocol, n (%)	Immediate 20 (15.6); Early 59 (46.1); Delayed 49 (38.3)	Immediate 57 (22.3); Early 99 (38.7); Delayed 100 (39.1)	χ²=3.03	0.220
Prosthesis type, n (%)	Single crown 83 (64.8); Bridge 34 (26.6); Full-arch 11 (8.6)	Single crown 196 (76.6); Bridge 56 (21.9); Full-arch 4 (1.6)	χ²=13.21	0.001
Occlusal overload, n (%)	24 (18.8)	18 (7.0)	χ²=12.03	<0.001
Occlusal adjustment during follow-up, n (%)	31 (24.2)	40 (15.6)	χ²=4.18	0.041
Maintenance visits per year, median [IQR]	2 [1–3]	3 [2–4]	Z=-3.83	<0.001
Plaque index, mean ± SD	1.80 ± 0.56	1.38 ± 0.47	t=7.46	<0.001

IQR, interquartile range; SLA, sandblasted, large-grit, acid-etched. Plaque index, maintenance frequency, and occlusal overload were restricted to documentation before the index/reference date. For variables with missing observations, descriptive summaries were averaged across the 20 imputed datasets; displayed categorical counts are rounded to the nearest whole number.

### 3.5 Case-only culture findings

Microbiological sampling was performed for all 128 peri-implantitis cases at the index presentation; controls were not sampled. Bacterial growth was recovered from 112/128 case episodes (87.5%), and 16/128 episodes (12.5%) were culture-negative. Culture-negative cases were retained in all matched clinical analyses but contributed no organism-level or susceptibility data. Submucosal plaque was the most common primary specimen (54/128, 42.2%), followed by peri-implant crevicular exudate (46/128, 35.9%) and purulent discharge (28/128, 21.9%) (**Table 3**). Mixed infections occurred in 40/128 cases (31.3%). Among organisms commonly discussed in relation to periodontal or peri-implant biofilms, *Porphyromonas gingivalis* and *Fusobacterium nucleatum* were recovered in 28.6% and 25.0% of culture-positive episodes, respectively. *Staphylococcus aureus* and *Escherichia coli* were also recovered (21.4% and 8.9%, respectively), but these are not characteristic microorganisms of peri-implantitis and are reported only as non-characteristic, potentially opportunistic culture findings rather than as representative disease-associated microbiota. The profile is limited to organisms cultivable under the conditions used, and these case-only frequencies cannot establish that any recovered organism was associated with peri-implantitis relative to health (**Supplementary Table 2**). Among isolates with interpretable antimicrobial susceptibility results, resistance patterns varied across antimicrobial categories; MRSA was identified in 6 of 24 *S. aureus* isolates (25.0%), and 20 of 160 isolates (12.5%) met the definition of multidrug resistance (**Supplementary Table 3**).

**Table 3:** Case-only specimen profile and culture yield among peri-implantitis episodes.

Item	n/N (%)
Specimen type (primary specimen), n (%)	
Submucosal plaque	54/128 (42.2)
Peri-implant crevicular exudate	46/128 (35.9)
Purulent discharge (pus)	28/128 (21.9)
Overall culture yield (any bacterial growth)	112/128 (87.5)
Culture-negative episodes	16/128 (12.5)
Culture positivity by specimen type	
Submucosal plaque	50/54 (92.6)
Peri-implant crevicular exudate	40/46 (87.0)
Purulent discharge (pus)	22/28 (78.6)

Culture yield is the proportion of 128 case episodes with any bacterial growth. The 16 culture-negative episodes were retained in all matched clinical analyses but contributed no organism-level or susceptibility data. Controls were not microbiologically sampled; all microbiological summaries are descriptive, and no organism-specific association with peri-implantitis was estimated.

### 3.6 Exploratory multivariable associations

The *post hoc* unconditional multivariable model retained seven temporally eligible indicators. History of periodontitis was associated with peri-implantitis (adjusted OR 1.93, 95% CI 1.25-2.99) and similar estimates across the missing-data and culture-positive sensitivity analyses. Immune-related disorders produced the largest point estimate (adjusted OR 5.84, 95% CI 1.67-20.47), but the wide interval reflects the small and heterogeneous exposed subgroup. Higher plaque index (adjusted OR 2.78, 95% CI 1.82-4.24), anodized surface (adjusted OR 2.16, 95% CI 1.41-3.32), pre-index occlusal overload (adjusted OR 2.08, 95% CI 1.14-3.78), and longer surgery duration (per 20 minutes: adjusted OR 1.14, 95% CI 1.05-1.24) were positively associated, whereas each additional annual maintenance visit was associated with lower odds (adjusted OR 0.80, 95% CI 0.70-0.92) (**Table 4**). All estimates remain exploratory because the covariate set was not prespecified.

**Table 4:** *Post hoc* exploratory unconditional multivariable logistic regression associations with peri-implantitis.

Indicator (coding)	Adjusted OR (95% CI)	Wald z	P value
History of periodontitis (yes vs no)	1.93 (1.25–2.99)	2.95	0.003
Immune-related disorders (yes vs no)	5.84 (1.67–20.47)	2.75	0.006
Anodized implant surface (yes vs no)*	2.16 (1.41–3.32)	3.55	<0.001
Surgery duration (per 20 min)	1.14 (1.05–1.24)	3.05	0.002
Pre-index occlusal overload (yes vs no)	2.08 (1.14–3.78)	2.38	0.017
Pre-index plaque index (per 1 unit)	2.78 (1.82–4.24)	4.79	<0.001
Pre-index maintenance visits (per 1 visit/year)	0.80 (0.70–0.92)	−3.10	0.002

*Reference category: non-anodized surfaces (including SLA and machined/other). Estimates are associative and not causal. The seven-variable covariate set was not prespecified; adjusted estimates and confidence intervals do not incorporate selection uncertainty. The model did not explicitly account for dependence within matched sets. Immune-related disorders were rare and heterogeneous, resulting in an imprecise estimate.

Abbreviations: CI, confidence interval; OR, odds ratio. No internal or external validation was performed.

### 3.7 Model diagnostics

Multicollinearity was evaluated using the variance inflation factor (VIF) for all covariates in the association model. VIF values ranged from 1.08 to 1.62 and were below the prespecified threshold of 5.0. The largest VIF was observed for plaque index (1.62), followed by maintenance visits (1.48), indicating no evidence of problematic multicollinearity. The seven-parameter model included 128 case events (18.3 events per parameter). Because the analysis was intended to estimate exploratory associations rather than to develop a prediction tool, no model-performance or validation claims are presented.

### 3.8 Missing data and sensitivity analyses

Missingness ranged from 0% to 6.8% across modeled variables and was quantified overall and compared between cases and controls (**Supplementary Table 4**). Effect directions for the seven indicators were similar across multiple imputation, complete-case, and missing-indicator analyses (**Supplementary Table 5**), and in the analysis restricted to culture-positive cases (**Supplementary Table 6**). These analyses assess sensitivity to missing-data handling and culture status; they do not constitute model validation.

## 4 Discussion

This study included a case-only culture description of 128 peri-implantitis episodes and a matched case-control analysis of clinical indicators. Bacterial growth was recovered in 112 episodes, whereas 16 were culture-negative. Because controls were not microbiologically sampled, the culture results are descriptive and cannot support comparisons between peri-implantitis and health. The matched analysis retained periodontitis history, immune-related disorders, implant surface, surgery duration, pre-index occlusal overload, plaque index, and maintenance attendance. These associations should be interpreted in the context of *post hoc* covariate selection, residual confounding, and the absence of model validation.

Among the retained indicators, history of periodontitis was associated with peri-implantitis (adjusted OR 1.93, 95% CI 1.25-2.99). The estimate was similar across complete-case, missing-indicator, and culture-positive sensitivity analyses (**Supplementary Tables 5**, **S6**), consistent with evidence that previous periodontal disease is associated with susceptibility to peri-implant inflammatory breakdown (Serroni et al., 2024). The association may reflect overlapping pathways, including a persistent dysbiotic reservoir in residual periodontal pockets, shared behavioral exposures, impaired plaque control, and an inflammatory host phenotype. Prospective and cross-sectional studies have likewise reported that systemic, behavioral, and periodontal characteristics contribute to variation in peri-implantitis occurrence (Romandini et al., 2021; Romandini et al., 2025). By contrast, immune-related disorders produced a larger point estimate but were present in only 13 participants and combined clinically heterogeneous conditions; the wide confidence interval indicates substantial uncertainty. Nevertheless, the periodontitis estimate arose from a *post hoc* observational model and remains susceptible to residual confounding, so it should not be interpreted as confirmatory or causal.

The associations involving anodized implant surface and longer operative duration require cautious interpretation. Experimental and translational literature indicates that surface chemistry and topography can influence microbial adhesion, biofilm formation, and host-tissue interactions (Gkioka and Rausch-Fan, 2024; Kadirvelu et al., 2024; Villegas et al., 2024). Nevertheless, implant surface is also linked to manufacturer, implant macrodesign, connection geometry, restorative protocol, and the period in which a system was adopted. The observed surface association may therefore represent residual confounding by implant system or treatment selection rather than a direct biological effect. Population-based analyses also show that peri-implantitis estimates depend strongly on treatment setting, case definition, and implant distribution (Derks et al., 2016). Operative duration may function as a proxy for surgical complexity, augmentation requirements, operator decisions, or cumulative opportunities for contamination; it should not be interpreted as an isolated modifiable exposure. Pre-index occlusal overload was also associated with peri-implantitis. Current clinical evidence supports biological plausibility but remains heterogeneous, while experimental work suggests that excessive loading can adversely affect peri-implant tissues under selected conditions (Miyata et al., 2000; Mojaver et al., 2025). Because overload was identified from clinical records rather than standardized instrumented measurements, misclassification and clinician-dependent assessment remain possible.

Plaque index and maintenance attendance were clinically relevant associations, although reverse causation remains possible. Higher pre-index plaque index was associated with increased odds of peri-implantitis, whereas more frequent maintenance visits were inversely associated. These findings accord with reviews emphasizing supportive peri-implant therapy, professional biofilm disruption, reinforcement of home-care practices, and individualized recall intervals (Grusovin et al., 2010; Araújo et al., 2024; Mojaver et al., 2026). Periodontal stability before implant treatment and structured follow-up after implantation may be particularly relevant for patients with a history of periodontitis or systemic vulnerability. Maintenance frequency may also reflect health literacy, adherence, socioeconomic access, and clinician concern. Early subclinical inflammation could increase or decrease attendance, and plaque measurements obtained shortly before diagnosis may partly reflect evolving disease. The temporal definitions used in the analysis excluded measurements recorded at or after the index presentation, but retrospective chart review cannot fully resolve this ambiguity. The reported odds ratios are therefore more appropriate for risk-oriented surveillance than for defining treatment thresholds or fixed recall schedules.

The microbiological findings should be interpreted strictly as a case-only, culture-dependent description of organisms that were viable and recoverable under the specified laboratory conditions. *Porphyromonas gingivalis* and *Fusobacterium nucleatum* were among the more frequently recovered taxa, whereas *Staphylococcus aureus* and *Escherichia coli* are not characteristic microorganisms of peri-implantitis. Their recovery may reflect opportunistic or transient colonization within individual diseased sites and should not be interpreted as defining or representative of peri-implantitis-associated microbiota. No single cultured organism predominated, and the absence of microbiological sampling in healthy controls precludes organism-specific comparisons with health. Mixed infections were frequent, consistent with the polymicrobial ecology reported in systematic reviews and sequencing studies (Carvalho et al., 2023). Sixteen episodes were culture-negative despite clinical peri-implantitis; possible explanations include low recoverable bacterial burden, prior antibiotic exposure, sampling or transport factors, and fastidious or uncultivable organisms. Culture-negative status therefore does not indicate absence of microorganisms. Although culture supports recovery of viable isolates and isolate-level susceptibility testing, it cannot detect nonviable organisms, organisms suppressed by transport or incubation conditions, or much of the fastidious/uncultivable community captured by culture-independent methods. Consequently, the reported profile should not be regarded as a complete characterization of the peri-implant microbiota.

Several limitations should be considered. First, the microbiological component was restricted to cases and relied on conventional culture. It therefore describes only organisms cultivable under the specified conditions and cannot characterize the complete peri-implant microbiota, establish that any recovered organism or resistance phenotype is associated with peri-implantitis relative to health, or infer absence of microorganisms from a culture-negative result. Second, retrospective documentation creates potential exposure misclassification, incomplete temporal information, and reverse causation, particularly for plaque, maintenance, antibiotic exposure, and occlusal variables. Third, matching balanced only index year and observation time and did not eliminate clinical confounding; the unconditional regression model did not explicitly account for dependence within matched sets, which may have affected standard-error estimates. Fourth, the seven-variable covariate set was not prespecified, and the reported confidence intervals do not incorporate selection uncertainty. Fifth, the immune-related category and some implant subgroups contained few exposed participants, so conventional maximum-likelihood estimates may be sensitive to small data changes. Sixth, peri-implantitis severity was not consistently staged across the study period, and pooling cases may have obscured stage-specific patterns. Seventh, the single-center sample may limit generalizability, particularly with respect to implant systems, prescribing practices, and local resistance patterns. Finally, no internal resampling or external validation was performed. Prospective studies incorporating microbiological sampling of diseased and healthy sites, prespecified covariates, matched-design-aware analyses, and independent validation cohorts are required to confirm the observed clinical associations.

## 5 Conclusion

This retrospective matched case-control study identified an association between history of periodontitis and peri-implantitis (adjusted OR 1.93, 95% CI 1.25-2.99). The other retained associations require cautious interpretation because of *post hoc* covariate selection, residual confounding, sparse exposures, and the absence of model validation. Culture findings describe only viable organisms recovered from peri-implantitis cases under the specified laboratory conditions and cannot be used to infer organism-disease associations because controls were not microbiologically sampled.

## Data Availability

The raw data supporting the conclusions of this article will be made available by the authors, without undue reservation.
