# Supplementary material for: Clinical factors associated with peri-implantitis and the culture-based microbiological profile of peri-implantitis cases: a retrospective matched case-control study
Source: Front Cell Infect Microbiol. 2026 Sep 24;16:1848675. doi: 10.3389/fcimb.2026.1848675 (PMC13645507; doi:10.3389/fcimb.2026.1848675)
Supplement: Supplementary file 1 [file Table1.docx]

Supplementary Table S1. Balance diagnostics and control-pool disposition before and after matching

| Variable | Unmatched Cases (n=128) | Unmatched Control Pool (n=936) | Unmatched Test Statistic | Unmatched P value | Unmatched SMD | Matched Cases (n=128) | Matched Controls (n=256) | Matched Test Statistic | Matched P value | Matched SMD |
| --- | --- | --- | --- | --- | --- | --- | --- | --- | --- | --- |
| Index year, n (%) |  |  | χ² = 6.56 | 0.161 | 0.244 |  |  | χ² = 0.00 | 1.000 | 0.000 |
| 2021 | 18 (14.1) | 220 (23.5) |  |  |  | 18 (14.1) | 36 (14.1) |  |  |  |
| 2022 | 30 (23.4) | 210 (22.4) |  |  |  | 30 (23.4) | 60 (23.4) |  |  |  |
| 2023 | 32 (25.0) | 190 (20.3) |  |  |  | 32 (25.0) | 64 (25.0) |  |  |  |
| 2024 | 28 (21.9) | 170 (18.2) |  |  |  | 28 (21.9) | 56 (21.9) |  |  |  |
| 2025 | 20 (15.6) | 146 (15.6) |  |  |  | 20 (15.6) | 40 (15.6) |  |  |  |
| Observation time to index/reference date, months (mean ± SD) | 14.2 ± 6.1 | 19.0 ± 8.5 | t = −7.91 | <0.001 | 0.649 | 14.2 ± 6.1 | 14.3 ± 6.0 | t = −0.15 | 0.879 | 0.017 |

SMD, standardized mean difference. The eligible control pool contained 936 patients; 256 were matched to all 128 cases and 680 eligible controls were not selected. Matching balanced index year and observation time only and should not be interpreted as controlling all clinical confounding. For index year, the maximum absolute category-level SMD is shown; |SMD| < 0.10 was considered acceptable.

Supplementary Table S2. Case-only culture-based organism profile among culture-positive peri-implantitis episodes

| Organism / grouping | n/N (%) |
| --- | --- |
| Organisms recovered at episode level |  |
| *Porphyromonas gingivalis* | 32 (28.6) |
| *Fusobacterium nucleatum* | 28 (25.0) |
| *Staphylococcus aureus*† | 24 (21.4) |
| *Streptococcus anginosus* group | 22 (19.6) |
| *Prevotella intermedia* | 20 (17.9) |
| *Aggregatibacter actinomycetemcomitans* | 16 (14.3) |
| *Pseudomonas aeruginosa* | 12 (10.7) |
| *Escherichia coli*† | 10 (8.9) |
| Mixed infection (≥2 bacterial species in an episode) | 40/128 (31.3) |
| Single-organism infection | 72/128 (56.3) |
| Culture-negative episodes | 16/128 (12.5) |

Episode-level presence indicates detection in an index peri-implantitis episode; percentages may exceed 100% because mixed infection was defined a priori as recovery of at least two distinct bacterial species from one episode. Organism-specific percentages use the 112 culture-positive episodes as the denominator. These frequencies are descriptive of cases only and cannot identify organisms associated with peri-implantitis because controls were not microbiologically sampled. The 16 culture-negative episodes were retained in the matched clinical analyses but contributed no organism-level data. † *Staphylococcus aureus* and *Escherichia coli* are listed because they were recovered in the case samples; neither is regarded here as a characteristic microorganism of peri-implantitis, and their detection should not be interpreted as representative of peri-implantitis-associated microbiota. The table reports only organisms cultivable under the specified sampling, transport, media, atmospheric, and incubation conditions and does not represent the complete peri-implant microbiota.

Supplementary Table S3. Case-only antimicrobial resistance summaries among isolates with interpretable susceptibility results from peri-implantitis episodes

| Antimicrobial category | Gram-positive resistant n/N (%) | Gram-negative resistant n/N (%) |
| --- | --- | --- |
| β-lactams | 18/60 (30.0) | 22/100 (22.0) |
| Fluoroquinolones | 12/60 (20.0) | 28/100 (28.0) |
| Macrolides | 20/60 (33.3) | 12/100 (12.0) |
| Tetracyclines | 10/60 (16.7) | 18/100 (18.0) |
| Lincosamides | 22/60 (36.7) | 15/100 (15.0) |
| MRSA among *S. aureus* | 6/24 (25.0) | — |
| MDR isolates (overall) | 20/160 (12.5) | — |

MDR, multidrug-resistant; MRSA, methicillin-resistant *Staphylococcus aureus*. Susceptibility categories followed the CLSI M100 edition in force at testing; MDR denotes acquired non-susceptibility to at least one agent in three or more antimicrobial categories. Denominators reflect isolates for which the corresponding antimicrobial category yielded an interpretable result. The denominator of 160 for MDR represents isolates with interpretable susceptibility results and is smaller than the total number of episode-level organism detections. These isolate-level summaries are descriptive of cultured organisms from cases only; the MRSA frequency is not evidence that *Staphylococcus aureus* or MRSA is associated with peri-implantitis.

Supplementary Table S4. Missingness of selected candidate and modeled variables in the matched cohort (n = 384)

| Variable | Missing, n/N (%) | Group comparison (Cases vs Controls) | P value |
| --- | --- | --- | --- |
| Age | 0/384 (0.0) | χ²=0.00 | 1.000 |
| Sex | 0/384 (0.0) | χ²=0.00 | 1.000 |
| BMI | 10/384 (2.6) | χ²=0.03 | 0.871 |
| Smoking status | 14/384 (3.6) | χ²=0.02 | 0.890 |
| Alcohol use | 8/384 (2.1) | χ²=0.02 | 0.879 |
| Diabetes mellitus | 0/384 (0.0) | χ²=0.00 | 1.000 |
| History of periodontitis | 0/384 (0.0) | χ²=0.00 | 1.000 |
| Immune-related disorders | 0/384 (0.0) | χ²=0.00 | 1.000 |
| Pre-index systemic antibiotic exposure | 12/384 (3.1) | χ²=0.12 | 0.733 |
| Implant region (anterior/posterior) | 0/384 (0.0) | χ²=0.00 | 1.000 |
| Implant surface category | 18/384 (4.7) | χ²=0.08 | 0.780 |
| Surgery duration | 16/384 (4.2) | χ²=0.05 | 0.821 |
| Occlusal overload | 20/384 (5.2) | χ²=0.16 | 0.689 |
| Maintenance visits/year | 22/384 (5.7) | χ²=0.10 | 0.752 |
| Plaque index | 26/384 (6.8) | χ²=0.13 | 0.722 |

BMI, body mass index. Missingness was quantified as n/N (%) overall and compared between cases and controls. The primary regression analysis used multiple imputation by chained equations. Descriptive summaries for variables with missing observations were averaged across the 20 imputed datasets; displayed categorical counts in the main tables are rounded to the nearest whole number.

Supplementary Table S5. Sensitivity analyses across missing-data handling strategies

| Indicator | Primary analysis (Multiple imputation), aOR (95% CI) | P value | Complete-case analysis, aOR (95% CI) | P value | Missing-indicator approach, aOR (95% CI) | P value |
| --- | --- | --- | --- | --- | --- | --- |
| History of periodontitis (yes vs no) | 1.93 (1.25–2.99) | 0.003 | 1.88 (1.18–3.00) | 0.008 | 1.91 (1.23–2.97) | 0.004 |
| Immune-related disorders (yes vs no) | 5.84 (1.67–20.47) | 0.006 | 5.30 (1.41–19.88) | 0.014 | 5.72 (1.61–20.34) | 0.007 |
| Anodized surface (yes vs no) | 2.16 (1.41–3.32) | <0.001 | 2.09 (1.32–3.31) | 0.002 | 2.12 (1.37–3.28) | 0.001 |
| Surgery duration (per 20 min) | 1.14 (1.05–1.24) | 0.002 | 1.13 (1.04–1.24) | 0.005 | 1.14 (1.05–1.24) | 0.003 |
| Occlusal overload (yes vs no) | 2.08 (1.14–3.78) | 0.017 | 1.98 (1.04–3.78) | 0.039 | 2.05 (1.12–3.76) | 0.019 |
| Plaque index (per 1 unit) | 2.78 (1.82–4.24) | <0.001 | 2.65 (1.66–4.22) | <0.001 | 2.74 (1.78–4.22) | <0.001 |
| Maintenance visits (per 1 visit/year) | 0.80 (0.70–0.92) | 0.002 | 0.81 (0.69–0.95) | 0.010 | 0.80 (0.69–0.93) | 0.003 |

aOR, adjusted odds ratio; CI, confidence interval. These analyses assess sensitivity to missing-data handling for the post hoc unconditional model but do not account for covariate-selection uncertainty or dependence within matched sets and do not constitute internal validation of model performance.

Supplementary Table S6. Sensitivity analysis restricted to culture-positive peri-implantitis cases

| Indicator | Culture-positive restriction, aOR (95% CI) | Wald z | P value |
| --- | --- | --- | --- |
| History of periodontitis | 1.86 (1.17–2.96) | 2.62 | 0.009 |
| Immune-related disorders | 5.41 (1.43–20.45) | 2.49 | 0.013 |
| Anodized surface | 2.08 (1.33–3.26) | 3.22 | 0.001 |
| Surgery duration (per 20 min) | 1.13 (1.04–1.23) | 2.89 | 0.004 |
| Occlusal overload | 1.98 (1.05–3.73) | 2.11 | 0.035 |
| Plaque index (per 1 unit) | 2.61 (1.68–4.05) | 4.30 | <0.001 |
| Maintenance visits (per 1 visit/year) | 0.82 (0.71–0.95) | −2.62 | 0.009 |

aOR, adjusted odds ratio; CI, confidence interval. Controls were retained from the matched clinical cohort; the restriction applied to cases only. This post hoc unconditional analysis does not address covariate-selection uncertainty, dependence within matched sets, or the absence of a healthy-implant microbiological comparator.

Supplementary Table S7. Detailed culture-isolate identification workflow and interpretive criteria

| Step | Procedure or criterion |
| --- | --- |
| Colony selection | All morphologically distinct colony types on primary plates were evaluated. One representative colony per morphotype and specimen was subcultured to purity; individual colonies within confluent growth were not each tested separately. |
| Initial phenotypic characterization | Colony morphology, Gram staining, aerotolerance, and organism-appropriate reactions, including catalase, oxidase, and coagulase, were assessed. Commercial biochemical panels were used when additional phenotypic resolution was required. |
| MALDI-TOF MS platform | All pure isolates were analyzed on a Microflex LT instrument using MALDI Biotyper software version 4.1 (Bruker Daltonics, Bremen, Germany). A direct-smear/formic-acid overlay was used initially; full extraction was performed when the first result did not reach the species-level threshold. |
| Identification thresholds | A Biotyper score ≥2.00 was accepted for species-level identification; 1.70–1.99 supported genus-level identification; <1.70 was considered unreliable. Isolates below 2.00 were re-extracted and retested. |
| Resolution of incomplete identification | Persistent genus-only results were reported at the highest taxonomic level supported by concordant morphology, Gram staining, and biochemical findings. Results remaining unreliable after repeat testing were not included in species-level summaries. |
| Episode-level reporting | An organism was counted once per index episode regardless of the number of colonies or repeat recovery on plates. Mixed infection denoted recovery of at least two distinct bacterial species from one episode. |
| Interpretive scope | The profile includes only viable organisms recovered under the specified culture conditions. No sequencing or other culture-independent profiling was performed; consequently, the findings do not represent the complete peri-implant microbiota. |
